# Draft Genome Sequences from *Cyclospora cayetanensis* Oocysts Purified from a Human Stool Sample

**DOI:** 10.1128/genomeA.01324-15

**Published:** 2015-11-19

**Authors:** Yvonne Qvarnstrom, Yuping Wei-Pridgeon, Wen Li, Fernanda S. Nascimento, Henry S. Bishop, Barbara L. Herwaldt, Delynn M. Moss, Vishal Nayak, Ganesh Srinivasamoorthy, Mili Sheth, Michael J. Arrowood

**Affiliations:** a Center for Global Health, Centers for Disease Control and Prevention, Atlanta, Georgia, USA; b IHRC, Inc., Atlanta, Georgia, USA; c National Center for Emerging and Zoonotic Infectious Diseases, Centers for Disease Control and Prevention, Atlanta, Georgia, USA

## Abstract

The parasite *Cyclospora cayetanensis* causes foodborne diarrheal illness. Here, we report draft genome sequences obtained from *C. cayetanensis* oocysts purified from a human stool sample. The genome assembly consists of 865 contigs with a total length of 44,563,857 bases. These sequences can facilitate the development of subtyping tools to aid outbreak investigations.

## GENOME ANNOUNCEMENT

*Cyclospora cayetanensis* is a coccidian parasite that causes cyclosporiasis. Humans become infected by ingesting food or water contaminated with mature (sporulated) oocysts. The most common symptom of infection is watery diarrhea (1). Cyclosporiasis is not thought to be endemic in the United States: the two established risk factors for U.S. cases are international travel to regions where cyclosporiasis is endemic and the consumption of contaminated fresh produce imported from such regions (1, 2). U.S. foodborne outbreaks have been detected almost every year since the mid-1990s (http://www.cdc.gov/parasites/cyclosporiasis/outbreaks/foodborneoutbreaks.html [1]). However, outbreak investigations and studies of the epidemiology of cyclosporiasis have been hampered by multiple factors, including the lack of laboratory methods for strain subtyping and for sensitive detection of oocysts in food and environmental samples. One approach for developing improved laboratory methods is to use genomics to identify potential genetic markers for parasite detection and subtyping. Since very limited genetic information is available, the objective of this study was to obtain whole-genome sequences of *C. cayetanensis*.

No methods are available for propagating this parasite in the laboratory (3). A human stool sample collected during an outbreak investigation in 2001 was selected for this study because it had a large number of oocysts (>10^7^). The stool sample had been stored in 2.5% potassium dichromate at 4°C since the time of collection and was identified as positive for *Cyclospora* sp. by UV fluorescence microscopy (4). The oocysts were purified from stool components using discontinuous sucrose and cesium chloride gradients, as described elsewhere (5). The highly autofluorescent oocysts were further purified by flow cytometry sorting (FACSAria III; BD Biosciences) and then by treatment with 50% household bleach for 10 min on ice. Genomic DNA was released from oocysts by 15 cycles of freeze-thaw incubations and purified using the DNeasy blood & tissue kit (Qiagen). Four aliquots of genomic DNA were sheared to average sizes of 453, 584, 862, and 1,054 bp, respectively, in an M220 ultrasonicator (Covaris). About 10 ng of each sheared DNA sample was used for library construction for Illumina sequencing using Ovation Ultralow library systems V2 (NuGEN). The barcoded libraries were subjected to sequencing using Illumina MiSeq reagent kits v2 (500 cycles) and v3 (600 cycles). A total of 147,760,794 reads were trimmed and assembled *de novo* using CLC Genomics Workbench 7.5.1 (CLC bio). The three best assemblies (the ones with the longest contig, the largest *N*_50_ value, and the lowest number of contigs, respectively) were merged using GAM-NGS (6) to enhance assembly contiguity and accuracy. The resulting assembly yielded 865 contigs with a total length of 44,563,857 bases, a G+C content of 51.9%, an *N*_50_ value of 187,023 bases, and a longest contig of 1,465,153 bases. The assembly included a full-length mitochondrial genome of 6,273 bases (contig 438) and two contigs comprising the apicoplast genome of 24,124 (contig 312) and 5,557 bases (contig 451), respectively.

### Nucleotide sequence accession numbers.

This whole-genome shotgun project has been deposited at DDBJ/EMBL/GenBank under the accession no. LIGJ00000000. The version described in this paper is the first version, LIGJ01000000.
